# The association between glycosylated haemoglobin and newly diagnosed hypertension in a non-diabetic Sudanese population: a cross-sectional study

**DOI:** 10.1186/s12872-022-02649-y

**Published:** 2022-05-10

**Authors:** Saeed M. Omar, Imad R. Musa, Omer Abdelbagi, Manal E. Sharif, Ishag Adam

**Affiliations:** 1 Faculty of Medicine, Gadarif University, Gadarif, Sudan; 2 Department of Medicine, Royal Commission Hospital in Al Jubail Industrial City, Al Jubail, Kingdom of Saudi Arabia; 3 Department of Pathology, AL Qunfudhah Faculty of Medicine, Umm Al-Qura University, Al Qunfudhah, Saudi Arabia; 4 College of Medicine, King Khalid University, Abha, Saudi Arabia; 5 Department of Obstetrics and Gynecology, Unaizah College of Medicine and Medical Sciences, Qassim University, Unaizah, Saudi Arabia

**Keywords:** Prevalence, Glycosylated haemoglobin, Hypertension, Associated factors, Sudan

## Abstract

**Background:**

Glycosylated haemoglobin (HbA1c) is considered reliable for diagnosing and monitoring diabetes mellitus (DM). It also indicates cardiovascular complications related to DM. However, only a few studies have been conducted on this topic.

**Methods:**

We conducted a cross-sectional study to investigate the association between newly diagnosed hypertension and HbA1c among non-diabetic Sudanese adults. The sociodemographic characteristics of the participants in the sample were gathered using a questionnaire, and HbA1c was measured using an Ichroma machine.

**Results:**

Three hundred and eighty-four healthy participants were enrolled in this study. The median (interquartile range [IQR]) age was 56.0 (14.0) years, and 72.1% of the participants were female. The median (IQR) body mass index (BMI) was 31.2 (8.7) kg/m^2^. One hundred and fifteen (29.9%) participants presented newly diagnosed hypertension. The results of the multivariate analysis showed that age (adjusted odd ratio [AOR] = 1.03; 95% confidence interval [CI] = 1.01‒1.05); BMI (AOR = 1.09; 95% CI = 1.05‒1.14); HbA1c levels (AOR = 2.18; 95% CI = 1.29‒3.67) was positively associated with newly diagnosed hypertension. For an HbA1c level of 5.0% or more, the sensitivity and specificity of newly diagnosed hypertension were 91.3% and 28.2%, respectively (area under the curve = 0.61; 95% CI = 0.55–0.67; *P* ˂ 0.001). Participants who presented HbA1c levels of 5.0% or more were found to be at higher risk for newly diagnosed hypertension (AOR = 2.53; 95% CI = 1.14‒5.61).

**Conclusion:**

The results of this study indicated a high prevalence of newly diagnosed hypertension, and HbA1c levels were positively associated with newly diagnosed hypertension.

## Introduction

The incidence of non-communicable diseases has increased globally, especially in African countries [1]. Non-communicable diseases are associated with significant morbidity, mortality and financial burdens [1]. Hypertension is a serious medical condition that is significantly associated with risks of heart, brain, kidney and other diseases [1]. Over one billion adults aged 30–79 years worldwide are expected to have hypertension, and low- and middle-income countries constitute a majority of this population [1]. The prevalence of hypertension was found to be 46% in adult Africans aged 25 and above, as compared to 35% in Europe and North America [1]. Several factors have been reported to be associated with hypertension, such as age [2], gender [3], obesity [4], smoking and alcohol consumption [5].

In 2019, the global prevalence of diabetes mellitus (DM) was estimated to be 9.3% (463 million people) and was expected to rise to 10.2% (578 million) and 10.9% (700 million) by 2030 and 2045, respectively [6]. Glycosylated haemoglobin (HbA1c) represents one of the most important diagnostic tools for the diagnosis of DM, and it is an indicator used to determine glycaemic control in most DM patients [7]. High baseline HbA1c levels have been predominantly associated with an increased incidence of hypertension independently of obesity and DM [8].

The current evidence of an association between HbA1c levels and hypertension is not conclusive. Although some previous studies have shown that HbA1c levels are associated with hypertension [3, 8–12], others have failed to identify a relationship between the two [13–16]. Moreover, HbA1c has prognostic importance because it is used to predict cardiovascular complications related to metabolic syndrome [8]. However, most previous studies on this topic have been conducted in high-income countries, and no data have been published on the association between HbA1c and hypertension in African populations. Therefore, it is vital to conduct such studies in Africa to yield the data necessary for carrying out evidence-based interventions. Among African countries, Sudan has been shown to have an estimated 30% prevalence of hypertension [17]. In 2019, the Diabetes Atlas, compiled by the International Diabetes Federation, included Sudan among countries that had a DM prevalence of more than 12% [6, 18]. We performed this study to assess the associations between HbA1c and newly diagnosed hypertension in eastern Sudan.

## Methods

### Study design and ethical approval

#### Study area

Gadarif, which is one of the 18 states of Sudan, has an area of 75,263 km^2^. Based on the 2008 census, the total population of this state is 1,336,662, and the annual population growth rate is 4.7% [19]. Collecting and trading forest products and charcoal burning are the traditional economic activities of the region, in addition to agriculture, grazing and forest utilisation. The livestock production carried out in the state serves to enhance the traditional pastoral and agropastoral systems [20].

A cross-sectional community-based study was conducted from January to May 2019, for which a multistage sampling approach was employed. We adopted simple random sampling to select four of the 11 localities (the smallest administrative units in Sudan) in Gadarif. In total, the sample comprised 384 participants, who were recruited from the four localities according to their size.

#### Inclusion criteria

Sudanese residents who were over 18 years of age and living in a household were chosen using the lottery method. The participants were apparently healthy subjects who were not known to have or be treated for DM or hypertension. If a selected house was not inhabited, if there was no suitable participant in the household or if the inhabitants refused to participate, we moved on to the next house.

#### Exclusion criteria

**Fig. 1:**
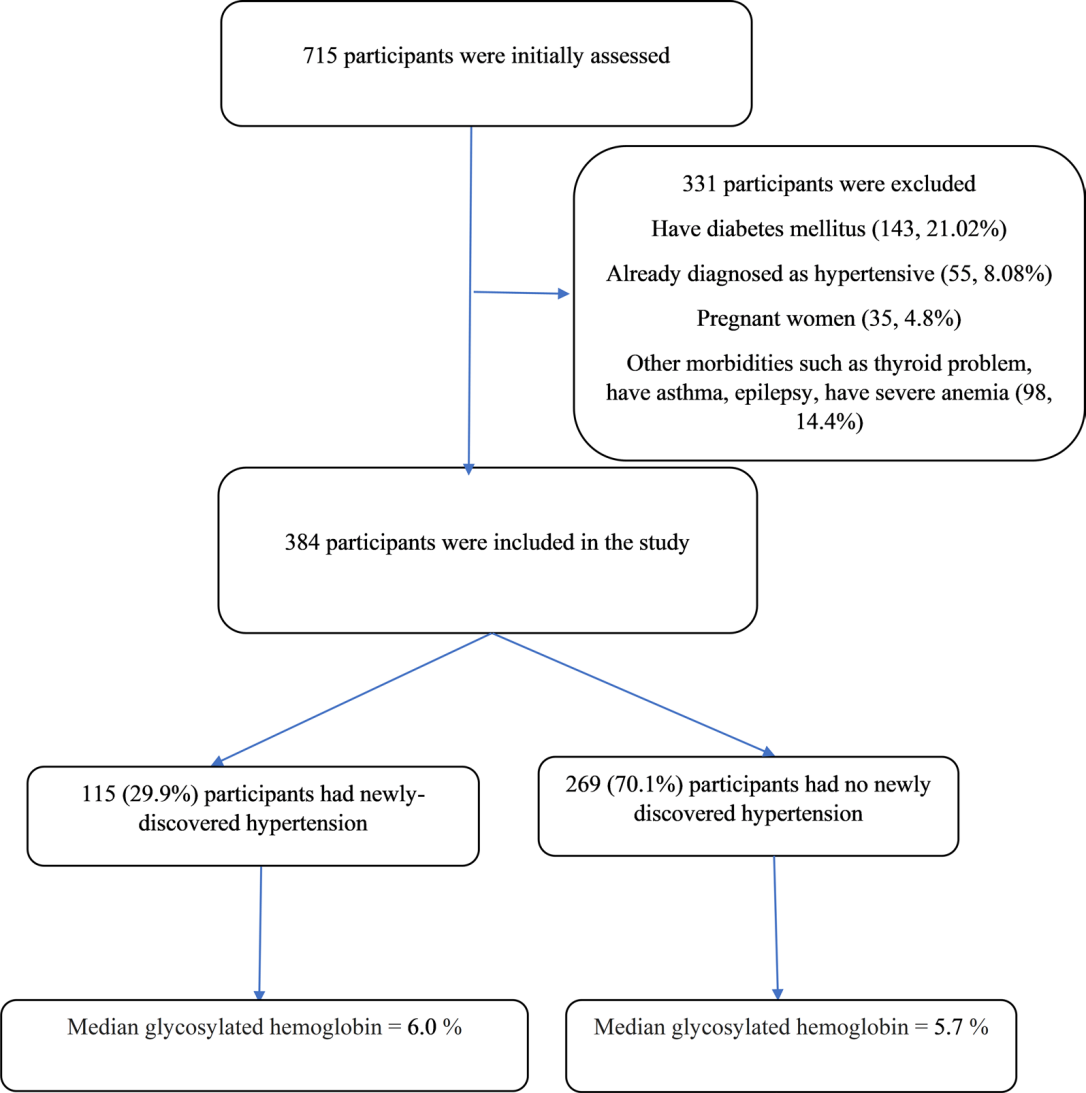
Shows the inclusion and exclusion criteria of the participants in eastern Sudan

The exclusion criteria were as follows: individuals below the age of 18, pregnant women, those with DM, hypertension, hemoglobinopathy, acute illnesses or psychosis, debilitated patients, those who refused to participate in the study, temporary residents and those suffering from any chronic disease that could affect HbA1c levels measurement (e.g., thyroid problems, asthma, epilepsy, severe anaemia or end-stage renal disease). Individuals who were on medications that affect blood sugar or blood pressure were also excluded (Fig. 1).

All eligible participants were invited to participate in the study, and two well-trained medical officers were assigned to interview those who agreed to do so. The study was explained to the participants, all of whom signed an informed consent form. The World Health Organisation’s three-level stepwise-approach questionnaire was adopted for data collection [21]. The questionnaire was employed to collect the following data: demographic and behavioural information and physical measurements, including anthropometric measurements, blood pressure and biochemical test results for non-communicable disease surveillance.

The questionnaire was used to collect the following sociodemographic characteristics: age, sex, marital status (married, divorced or unmarried), employment status (employed or unemployed), education level (less than secondary level or equal to or higher than secondary level), smoking habits (those who had smoked more than 100 cigarettes in their lives and reported having smoked in the past year were considered smokers) and alcohol consumption (one or more drinks in the previous month).

The weight and height of the participants were measured using standard procedures. Next, their body mass index (BMI) was obtained using the following equation: weight (kg)/height (m^2^). Their blood pressure was measured using a standard mercury sphygmomanometer after the patients had rested for at least 10 min in a seated position. The procedure was fully explained to each participant before their arm was maintained at the level of the heart using an appropriately sized cuff. The mean of two (at an interval of 1–2 min) blood pressure readings was recorded. If there was a difference of more than 5 mmHg between the two readings, the measurements were taken again until a stable reading was achieved.

#### Blood glucose measurement

A total of 3 ml of venous blood was drawn from each participant after providing a thorough explanation of the associated procedure and technique. The blood samples were collected under aseptic conditions in a vacuum blood collection tube containing ethylene diamine tetraacetic acid (EDTA). Random blood glucose levels were immediately tested for the samples using a glucometer (Accu-Check Active, Roche Diagnostics, Germany). The samples were transported to a modern diagnostic laboratory where HbA1c levels were measured using an Ichroma machine (Republic of Korea).

#### Diagnosis of DM

DM was defined as recommended by the American Diabetes Association and the International Diabetes Federation for non-pregnant adults (HbA1c levels of 6.5% or higher) [18, 22]. We selected the HbA1c test for this study because it is convenient to administer and evaluate. Moreover, it reflects the average blood glucose levels during the previous two to three months and directly correlates to blood glucose levels. The HbA1c test is practical, and, as it is not affected by recent food intake, a fasting period is not required. Further, it can be administered at any time of the day.

#### Diagnosis of hypertension

The participants were considered hypertensive if their systolic blood pressure was 140 mmHg or more, their diastolic blood pressure was 90 mmHg or more or both these criteria were met in both repeated measurements [23].

#### Sample size calculation

A single population proportion Kish Leslie formula (n = z^2^ *p* (1—*p*)/d^2^) was used to calculate the sample size. We assumed that the maximum proportion (50%) of newly discovered hypertension had the maximum sample size estimation. Thus, our sample of 384 participants presented a 95% confidence level, and we expected that 10% of them might not respond or might have incomplete data.

### Statistical analysis

The data were analysed using SPSS for Windows (version 22.0). Continuous data (including HbA1c) were checked for normality using the Shapiro–Wilk test, and it was found that all data were not distributed normally. The data were expressed as a numerical proportion or as a median (interquartile range [IQR]) where applicable. A univariate analysis was performed, in which newly diagnosed hypertension was the dependent variable. The independent variables were age, gender, employment, education, BMI and HbA1c. Multicollinearity (variance inflation factor pf less than four) was checked but not detected. A logistic regression analysis was applied to the variables if their univariate *p* was less than 0.20, and a backward-stepwise likelihood ratio regression was used for adjustment. Next, the odds ratios (OR) and 95% confidence intervals (CI) were calculated. A *p* value of less than 0.05 was considered significant. Reliability tests (sensitivity and specificity) and cut-off values for HbA1c were conducted using the receiver operating characteristic (ROC) curve and the area under the curve (AUC).

## Results

**Fig. 2:**
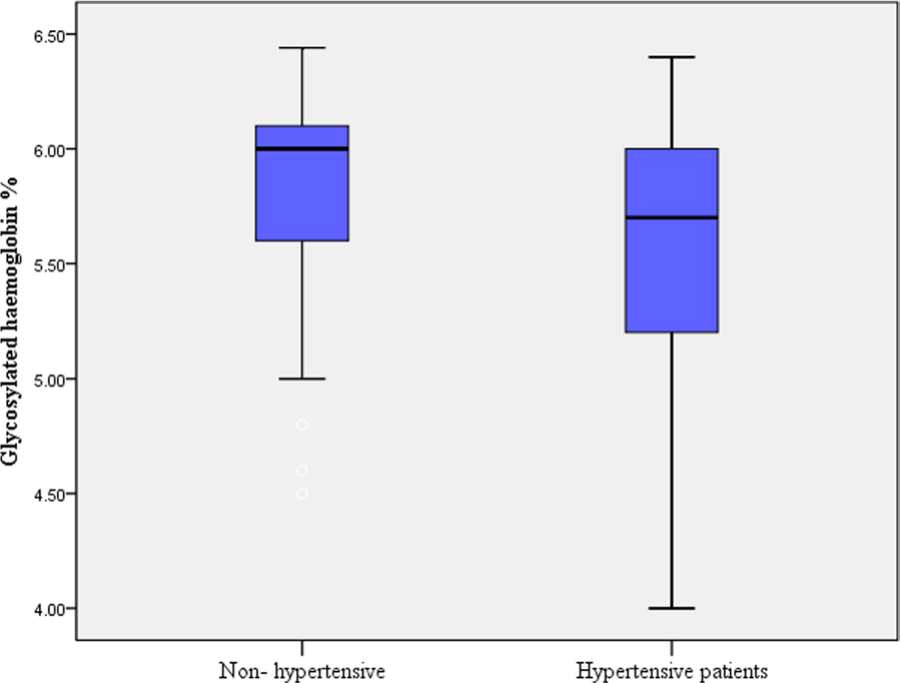
Box plot of HbA1c levels among hypertensive and non-hypertensive non-diabetic subjects

Initially, 715 participants were screened, of which 331 were excluded for certain reasons such as DM, pregnancy and medical diseases (Fig. 1). Thus, 384 healthy non-diabetic participants were enrolled in the study. The median (IQR) age was 56.0 (14.0) years, and 329 (72.1%) participants were female. Of these, 237 (61.7%) were married, 239 (62.2%) had above secondary level education and 124 (32.3%) were employed. Only 7 (1.8%) participants were smokers, and none of them were alcoholics. The median (IQR) BMI was 31.2 (8.7) kg/m^2^ (Table 1). Finally, 115 (29.9%) participants were found to have newly diagnosed hypertension. The HbA1c levels were significantly higher among patients with newly diagnosed hypertension compared to those who were non-hypertensive (Fig. 2).

**Table 1:** Demographic and physical characteristics of the investigated participants (N = 384) in eastern Sudan, 2020

Variables	Median	Interquartile range
Age, years	56.0	14.0
Body mass index, kg/m^2^	31.2	8.7
Glycosylated hemoglobin, %	Number	Proportion
*Gender*		
Female	277	72.1
Male	107	27.9
*Marital*		
Married	237	61.7
Single/divorced	147	38.3
*Education*		
≥ Secondary level	239	62.2
˂ Secondary level	145	37.8
*Employment*		
Yes	124	32.3
No	260	67.3
*Smoking*		
No	377	98.2
Yes	7	1.8

The univariate analysis included patients with newly diagnosed hypertension who were significantly older, had high levels of HbA1c, were married, had above secondary education and were obese (Table 2).

**Table 2:** Univariate analysis of the factors associated with hypertension in eastern Sudan, 2020

Variables	Hypertensive (n = 115)	Non-hypertensive (n = 269)	OR (95.0%CI)	*P*
	Median (IQR)	Median (IQR)		
Age, years	42.0 (26.0)	32.0 (19.0)	1.01(1.01‒1.04	< 0.001
Body mass index, kg/m^2^	28.5 (11.2)	24.1(6.7)	1.10 (1.06‒1.14	< 0.001
Glycosylated hemoglobin, %	6.0 (0.5)	5.7 (0.8)	2.32(1.41‒3.79	0.001
	N (proportion)	N (proportion)		
*Gender*				
Female	78 (67.8)	199 (74.0)	0.74 (0.46‒1.19)	0.219
Male	37 (32.2)	70 (26.0)	Reference	
*Marital*				
Single/divorced	33 (28.7)	114 (42.4)	0.54 (0.34‒0.87)	0.012
Married	82 (71.2)	155 (57.6)	Reference	
*Education*				
> Secondary level	59 (51.3)	180 (66.9)	Reference	0.012
≤ Secondary level	56 (48.7)	89 (33.1)	1.92 (1.23‒2.99)	
*Employment*				
Yes	39 (33.9)	85 (31.6)	Reference	0.657
No	76 (66.1)	184 (68.4)	0.90 (0.56‒1.43)	

IQR = Interquartile range, OR = odds ratio, CI = confidence interval, N = number

The results of the multivariate regression analysis were as follows: age (AOR = 1.03; 95% CI = 1.01‒1.05), BMI (AOR = 1.09; 95% CI = 1.05‒1.14) and HbA1c levels (AOR = 2.18; 95% CI = 1.29‒3.67) were found to be positively associated with newly diagnosed hypertension. There was no significant association between marital status, level of education and newly diagnosed hypertension (Table 3).

**Table 3:** Multivariate analysis of the factors associated with hypertension in eastern Sudan, 2020

	Non-adjusted		Non-adjusted	
Variables	OR (95.0% CI)	*P*	OR (95.0% CI)	*P*
Age, years	1.02 (1.01‒1.04)	< 0.001	1.03 (1.01‒1.05)	< 0.001
Body mass index, kg/m^2^	1.09 (1.05‒1.14)	< 0.001	1.09 (1.05‒1.14)	< 0.001
Glycosylated hemoglobin, %*	2.02 (1.27‒3.64)	0.004	2.18 (1.29‒3.67)	0.003
*Marital*				
Single/divorced	0.84 (0.50‒1.42)	0.531		
Married	Reference			
*Education*				
> Secondary level	Reference	0.144		
≤ Secondary level	1.47 (0.88‒2.43)			
Glycosylated hemoglobin, %*				
≥ 5.0%	2.47 (1.10‒5.54)	0.027	2.53(1.14‒5.61)	0.022
˂ 5.0%	Reference		Reference	

^*^Were entered one by one. IQR = Interquartile range, OR = odds ratio, CI = confidence interval

**Fig. 3:**
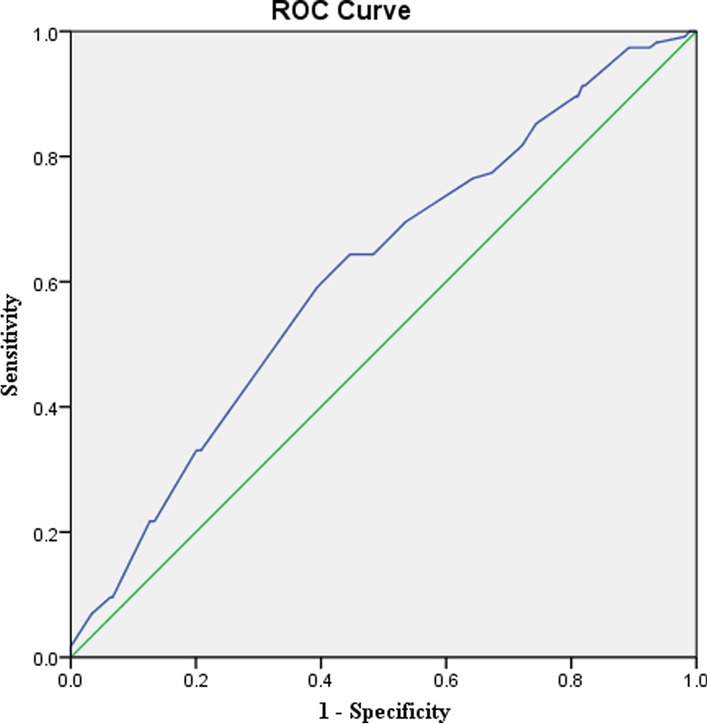
ROC curve analysis of HbA1c and hypertension risk in non-diabetic subjects

For HbA1c levels of 5.0% and above, the sensitivity and specificity of the diagnosis of newly diagnosed hypertension were 91.3% and 28.2%, respectively (AUC = 0.61; 95% CI = 0.55–0.67; *P* ˂ 0.001) (Fig. 3). Thus, participants with HbA1c levels of 5.0% or more were at higher risk for newly diagnosed hypertension (AOR = 2.53; 95% CI = 1.14‒5.61; *P* = 0.022).

## Discussion

The main finding of our study was the association between HbA1c levels and newly diagnosed hypertension. In this study, each percentage increase in HbA1c was observed to increase the risk for newly diagnosed hypertension by 2.18 times (AOR = 2.18). Using a cut-off point for HbA1c levels of 5.0% or more, the sensitivity and specificity of the diagnosis of hypertension were calculated as 91.3% and 28.2%, respectively. These results are in line with those of a prospective cohort study that includes 9,603 middle-aged American participants, where elevated HbA1c levels (even without a prior diagnosis of DM) were associated with hypertension among diabetic as well as non-diabetic individuals [8]. In a previous study with 19,858 non-diabetic American women, the highest HbA1c quintile was associated with hypertension among the participants [10]. Further, the results of the Framingham Heart Study also revealed that high HbA1c levels were associated with hypertension [9]. A recent study on 376,644 cases in the United Kingdom found that HbA1c was a significant predictor of hypertension risk [11]. Two studies conducted in China showed that higher HbA1c levels significantly increased the risk of hypertension and isolated systolic hypertension among non-diabetic patients without significantly affecting diastolic blood pressure [3, 12]. Additionally, a significant association was observed between HbA1c and hypertension among patients with T2DM in Brazil [24, 25], Korea [26] and Belgium [27].

In contrast to our findings, a recently published study demonstrated that HbA1c in diabetic patients was not correlated with arterial stiffness; however, the concomitant hypertension was a significant potential risk for arterial stiffness [13]. Furthermore, certain studies demonstrated a non-significant association between HbA1c and the risk of developing hypertension in non-diabetic populations in Israel [14], Japan [15] and Germany [16]. Another study reported a non-significant association between HbA1c and the increased risk of developing hypertension among women without diabetes after adjusting for BMI [10]. Interestingly, a non-significant correlation was found between the HbA1c levels of patients with T2DM and the risk of them developing either systolic or diastolic blood pressure in Iran [28, 29] and Brazil [30]. Surprisingly, two previous studies reported an inverse relationship between HbA1c levels and high blood pressure in patients with T2DM [31, 32].

Insulin resistance is the main pathophysiological factor behind the increased risk of developing both T2DM and hypertension [33]. In addition, the coincidence of glycaemic control and hypertension can be explained by dysfunctional pancreatic β cells and adiposity along with insulin resistance [8, 34]. HbA1c is one of the best indices for assessing insulin resistance in not only diabetic patients but also obese non-diabetic individuals [35]. Insulin resistance was shown to be dramatically upregulated across the quartile levels of HbA1c in those without diabetes [36]. High levels of HbA1c have been linked to proinflammatory cell signalling and oxidative stress, which may induce arterial stiffness [37]. Similarly, higher HbA1c values were shown to predict the development of hypertension as a result of high glucose flux across endothelial cell membranes [38]. Furthermore, the formation and accumulation of advanced glycation end products was observed to stem from excess circulating glucose that binds proteins, lipids and nucleic acids [39]. The accumulation of advanced glycation end products in the vessel wall was indicated to be the main contributor for the inflammation process, oxidative stress and endothelial dysfunction, subsequently altering blood pressure [39], increasing systemic vascular resistance and causing arterial stiffness [30]. In addition, the glycosylation of haemoglobin was demonstrated to impair oxygen carrying capacity, hence promoting hypoxia and the associated systemic vascular vasodilatory adaptations and inflammatory responses [40]. Similarly, increased levels of HbA1c were observed to facilitate endothelial damage, which led to the further release of endothelin from the endothelial cells and hindered the production of prostacyclin and nitric oxide, resulting in vasomotor dysfunction and further elevation in blood pressure [41]. In the current study, greater age was statistically significantly associated with the risk of developing hypertension. This result is consistent with that of a previous study conducted in Sudan, which revealed a significant association between increasing age and the prevalence of hypertension [2]. Similarly, a recent meta-analysis that included 43,025 older African adults (above the age of 53) demonstrated that older age was independently associated with hypertension [42]. Our results indicated that the BMI is a significant predictor of the development of hypertension. Several previous studies have illustrated significant associations between obesity and hypertension in Africa [5, 42–45]. The results of the current study and a recently published paper in Sudan showed no significant association between an individual’s gender and the presence of hypertension [2]. However, some previous studies reported that gender was a significant predictor of hypertension [3, 46]. Interestingly, in the present study, only 1.8% of the participants were smokers, and no participants reported alcohol consumption. It is possible that the rates of smoking and alcohol consumption were underestimated, as many individuals may not have been willing to admit these social habits given that they are stigmatised, particularly among females.

## Conclusion

HbA1C is a significant predictor of newly diagnosed hypertension among healthy adult non-diabetic Sudanese. Hence, it can be used to modify or prevent hypertension among those who have considerably high HbA1c levels.

### Limitations of the study

Our study has the following limitations. The causes and the effects of the results could not be inferred, as this was a cross-sectional study. Only a single measurement of HbA1c at baseline was available, which may have resulted in misclassification. Other causes of secondary hypertension cannot be excluded. Moreover, other factors, such as dyslipidemia, were not assessed. Also the high number of females participant which may be due to the availability of females at home during the data collection.

## Data Availability

The datasets used and/or analysed during the current study are available in the [OSF] repository**,** [https://osf.io/jcx6b/?view_only=3ffb18e18d804929ab2008377924d572].
